# A call for diversity, equity, and inclusion: Highlights from the Consortium of Universities for Global Health 2021 conference

**DOI:** 10.1371/journal.pmed.1003607

**Published:** 2021-04-15

**Authors:** Beryne Odeny

**Affiliations:** Public Library of Science, San Francisco, California, United States of America and Cambridge, United Kingdom

## Abstract

Beryne Odeny reports from the CUGH 2021 virtual conference.

The first virtual Consortium of Universities for Global Health (CUGH) 2021 conference was held in March, 2021 [1]. Two weeks of satellite symposia culminated in this highly prestigious conference, which drew an eclectic group of renowned speakers, global health leaders, program implementers, researchers, and students from across the globe. There were more than 5000 delegates from diverse disciplines including public health, politics, education, medicine, planetary health, and finance. Top of the agenda was addressing critical gaps in global health and development against the backdrop of the COVID-19 pandemic.

CUGH is an organization of over 170 academic institutions and organizations throughout the world, engaged in addressing global health challenges [1]. The 2021 conference was meticulously and creatively planned as was evidenced by the dynamic virtual platform, which hosted several global leader interviews, general sessions, 40 concurrent sessions, 7 plenary sessions, over 700 poster programs, and the Pulitzer Center Film festivals–yes, movies were on the menu [2]. Best of all, the platform held up, with minimal technical difficulties. The conference agenda had curated sessions carefully customized to varying attendee interests and expertise. Participants could seamlessly and discreetly shuttle between sessions.

The inaugural interviews, with Dr. Anthony Fauci of the United States and Dr. Hugo Lopez-Gatell of Mexico, set the tone with emphasis on a much-needed global response to the ongoing pandemic. “2020 was a watershed moment in Global Health,” said Dr. Fauci. The COVID-19 pandemic indiscriminately unveiled the fragility of health systems in high income countries (HIC) and low- and middle-income countries (LMICs) alike. He unpacked the origins, evolution, and contention around current public health mandates such as mask wearing. He discussed vaccines–exploring vaccine manufacturing in LMICs, open patents, implications of emerging COVID-19 variants, and advice on curbing the prevailing vaccine infodemic (i.e., pandemic of misinformation) [2–4]. Dr. Lopez-Gatell described the pandemic as a “massive social event” fueled by deficits in health systems, politics, and governance, and by the growing tide of non-communicable diseases (NCDs) [5]. In a brief video recording, Dr. Tedros Adhanom Ghebreyesus, WHO’s Director-General, implored global partners to sign the COVID-19 Declaration on vaccine equity which he termed “the defining challenge of 2021” [6].

The post-pandemic forecast for global health was dire. COVID-19 has disrupted decades of progress toward attainment of Universal Health Care (UHC) and it will be doubly difficult to restore, by 2035, health indicators to their levels prior to the pandemic [7–9]. A modelling study by Dr. Wenhui Mao of Duke University showed that, even in the most optimistic scenario, it may not be possible to achieve UHC in the next decade without breakthrough technologies and exceptional political commitment. Among four critical indicators of TB mortality rate, HIV mortality rate, under 5 mortality ratio, and maternal mortality ratio, Dr. Mao found that only the HIV indicator had potential for recovery by 2035.

The metaphorical elephant in the room, and now its opposite, “the elephant not in the room”, respectively encapsulate two themes: neocolonialism and equity, especially for marginalized groups. Neocolonialism–a progeny of colonialism–resulting from sustained global North-South power imbalances, manifests in low prioritization of the most pressing challenges and diseases in LMICs. Equity was a poignant theme across the CUGH sessions and satellite symposia. Sessions were dedicated to exploring the hegemonic structures and institutional systems that underpin adverse health system performance and outcomes. A sampling of wide-ranging topics on global challenges exacerbated by neocolonialism and inequities comprised: a) elevating the visibility and power of researchers in LMICs, including fragile and conflict affected settings, through equitable access to funding, research autonomy and leadership, access to scholarly publishing, and senior authorship of research articles [10]; b) training next-generation global health professionals and building capacity for resource-challenged settings to address NCDs, including cancer care [5]; c) the Latin American and Caribbean health crises drawn by social gradients and inequities; d) navigating conflicting interests between public health and the corporate food industry; e) the dearth and role of women leaders in global health and in the COVID-19 response; f) the disproportionate incidence of HIV in adolescent girls and young women in sub-Saharan Africa (SSA) [11]; g) the disparate burden of neonatal mortality in LMICs and marginalized communities within HIC; and h) leveraging the power of film to evoke emotion and induce a consolidated response to global challenges. In addition, various facets of the human ecosystem were unpacked including climate change, biodiversity preservation, political climate, and the global kleptocracy, with attention to their implications for the health of the most marginalized populations.

Despite the highlighted issues, there is, potentially, a panacea for these inequities and challenges. One speaker, Dr. Lisa Adams of Dartmouth College, proposed a paradigm shift that summarized a wide range of deliberations–“moving global health out of the realm of charity into global citizenship, security, human rights, equal partnership, and interdisciplinary collaboration between LMICs and HICs.” Moving forward, more deliberate effort should be given to some elements. First, rethinking governance and funding at a global level while promoting the autonomy of LMICs and conflict-affected settings to drive their health agenda–independent from HIC interests. Bringing the elephant into the room by making equal space for LMICs to set the agenda at global tables of discussion around funding, research, and development will be pivotal to dismantling neocolonialism. Furthermore, funders and partners should work with in-country systems in LMICs as opposed to bypassing them. This is essential to building resilient health systems unified at national levels to allow for cross-discipline collaborations and swift responses to health threats. Rwanda is a laudable example, having swiftly remodeled its existing health systems including routine electronic information systems for nationwide COVID-19 surveillance, testing, contact tracing, and vaccination. Second, investing time to build trusting relationships between researchers or implementers and policy makers by upholding a participatory approach to research and implementation of evidence-based practices. This is essential globally, to support development of global public goods such as vaccines, free from market dynamics and aimed at universal and equitable access. Third, introduce policies that engage economies to produce with less fragmentation of nature and reduced pollution. These include protected area management, financing of nature-positive projects, and conservationist work for natural capital preservation. Global and public health practitioners need to educate and empower citizens to choose healthy and ecologically sustainable consumption practices. Fourth, promoting development of novel technologies for preventing HIV infection, such as broadly neutralizing antibodies, could overturn the unequal burden of HIV in adolescents and young women in SSA. Finally, HIC have a lot they can learn from LMICs. COVID-19 evidently demonstrated that a country’s Global Health Security Index ranking is not necessarily commensurate to its degree of success in handling pandemics, among other public health threats [8,12,13].

Throughout the conference, it was apparent that equity and collectivity in global health are necessary–not optional. Dr. Elvin Geng of Washington University, St. Louis remarked that the path to equity should be measurable with routinely incorporated metrics that track interventions to redress inequity and foster accountability. To achieve this, the tools of implementation science can be employed at both regional and global levels [14]. Overall, the remarkable interlacing of diverse disciplinary sessions at CUGH 2021 not only brought to light pressing world problems but equipped participants with a wellspring of potential remedies and collaborative opportunities. The panelists and speakers effectively portrayed the layered and multidimensional nature of global challenges underscoring the need for similarly multifaceted solutions. CUGH 2021 sparked thought-provoking discourse around global health strategies and re-invigorated the collective passion of global health experts, novices, and everyone in between, to build forward better.
